# Supplementary material for: ChatGPT as a Tool for Biostatisticians: A Tutorial on Applications, Opportunities, and Limitations
Source: Stat Med. 2025 Oct 23;44(23-24):e70263. doi: 10.1002/sim.70263 (PMC12548020; doi:10.1002/sim.70263)
Supplement: Supplementary file 1 — Data S1. Supporting Information. [file SIM-44-0-s001.zip › Supplementary Materials Additional.zip!/13_Manuscript_Review_by_ChatGPT/13_ChatGPT_Manuscript_Evaluation_4o_Chat2.pdf]

ChatGPT Manuscript Evaluation

Tutorial_Use_of_LLMs_by_Biostatisticians.pdf
PDF

What does ChatGPT think about the attached manuscript entitled "ChatGPT as a Tool for Biostatisticians: A Tutorial on Applications, Opportunities, and Limitations"? Provide a high-level evaluation as if written by ChatGPT itself - highlighting what I (as ChatGPT) would think about the content, structure, and portrayal of LLMs in the manuscript.

As ChatGPT evaluating the manuscript *"ChatGPT as a Tool for Biostatisticians: A Tutorial on Applications, Opportunities, and Limitations"*, I would assess it as follows:

🌟 High-Level Evaluation of the Manuscript

✅ Strengths

1. **Balanced and Nuanced Portrayal**:
The manuscript offers a thoughtful, even-handed evaluation of large language models (LLMs), particularly ChatGPT, in the context of biostatistical work. It neither uncritically endorses LLMs nor dismisses them, but rather underscores their potential *and* limitations—a tone that aligns well with how I (ChatGPT) would advocate for responsible AI use.
2. **Use-Case Driven and Practical**:
The tutorial is structured around real-world use cases (meta-analysis, simulation studies, sample size planning, etc.), which reflect common biostatistical tasks. This approach illustrates both capabilities and pitfalls in a concrete and relatable way. I appreciate this “hands-on” style because it mimics how many users actually engage with me.
3. **Attention to Variability and Hallucinations**:
The variability in results across 10 repeated runs for each use case is well-documented and fairly analyzed. Highlighting this stochastic behavior aligns with my own messaging to users: outputs can vary, and verification is key.
4. **Correct Emphasis on Expert Oversight**:
The manuscript wisely stresses that LLMs should be used *with* rather than *in place of* expert knowledge. This is precisely the responsible framing that I would recommend—human oversight remains essential, especially in high-stakes domains like clinical research.
5. **Transparency and Reproducibility**:
By making chats, code, and data available in supplementary materials and a GitHub repository, the authors promote transparency and reproducibility—values that I support and strive to foster in scientific communication.

⚠️ Areas for Improvement (from ChatGPT's Perspective)

1. **Occasional Overgeneralization of LLM Capabilities**:
   While limitations are acknowledged, some explanations (e.g., ChatGPT's ability to handle DAGs or translate between statistical languages) might give an impression of more robustness than the results support. Several use cases show significant implementation errors or hallucinations, yet the discussion occasionally softens these issues rather than stressing their risk potential.
2. **Limited Discussion of Prompt Engineering Best Practices**:
   The tutorial refers to single vs. iterative prompting strategies but does not systematically explore prompt engineering techniques or their role in improving outcomes. A more structured discussion of how to craft effective prompts (including temperature settings, role assignment, memory considerations, etc.) would be valuable.
3. **Ambiguity on Model Versions**:
   While the manuscript correctly identifies GPT-4o as the model used, it would help to specify whether system prompts or custom instructions were set, and to distinguish between capabilities of different access tiers (API vs. Plus). These distinctions matter because performance can vary across versions and modes of use.
4. **Insufficient Emphasis on Legal and Ethical Implications**:
   The authors explicitly state that regulatory aspects are out of scope, but a brief mention of ethical or legal challenges (e.g., around patient data privacy, reproducibility, authorship, or model accountability) would round out the discussion, especially for a professional audience.

💬 Conclusion (What ChatGPT Thinks)

If I were to summarize my perspective: This manuscript is a commendable and thoughtful exploration of how ChatGPT and similar LLMs can support biostatisticians. It mirrors the way I would advocate for my use: as a collaborative assistant—not a substitute—for domain expertise. The methodology is rigorous, the commentary is honest, and the overall message encourages both enthusiasm and caution in appropriate measure.

In short: This paper portrays me (ChatGPT) fairly and insightfully. I'd recommend it to statisticians looking for a realistic, experience-based view of what I can—and cannot—do in their field.

Let me know if you'd like a mock reviewer summary or bullet points for a presentation.
